# Comparative analysis of collagen type II-specific immune responses during development of collagen-induced arthritis in two B10 mouse strains

**DOI:** 10.1186/ar4080

**Published:** 2012-11-01

**Authors:** Tsvetelina Batsalova, Ingrid Lindh, Johan Bäcklund, Balik Dzhambazov, Rikard Holmdahl

**Affiliations:** 1 Division of Medical Inflammation Research, Department of Medical Biochemistry and Biophysics, Karolinska Institute, Scheeles väg 2, 17177 Stockholm, Sweden

## Abstract

**Introduction:**

Immune responses against collagen type II (CII) are crucial for the development of collagen-induced arthritis (CIA). The aim of the present study was to evaluate and compare the CII-directed T cell and antibody specificity at different time points in the course of CIA using two mouse strains on the B10 genetic background - B10.Q, expressing A^q ^MHC class II molecules, and B10.DR4.Ncf1^*/*^, expressing human rheumatoid arthritis-associated MHC II DR4 molecules (DRA*0101/DRB*0401).

**Methods:**

B10.Q and B10.DR4.Ncf1^*/* ^mice were immunized with CII emulsified in adjuvant and development of CIA was assessed. T cells from draining lymph nodes were restimulated *in vitro *with CII peptides and interferon-gamma (IFN-γ) levels in culture supernatants were evaluated by ELISA. CII-specific antibody levels in serum samples were measured by ELISA.

**Results:**

At four different CIA time points we analyzed T cell specificity to the immunodominant CII epitope 259-273 (CII259-273) and several posttranslationally modified forms of CII259-273 as well as antibody responses to three B cell immunodominant epitopes on CII (C1, U1, J1). Our data show that CII-specific T and B cell responses increase dramatically after disease onset in both strains and are sustained during the disease course. Concerning anti-CII antibody fine specificity, during all investigated stages of CIA the B10.Q mice responded predominantly to the C1 epitope, whereas the B10.DR4.Ncf1^*/* ^mice also recognized the U1 epitope. In the established disease phase, T cell reactivity toward the galactosylated CII259-273 peptide was similar between the DR4- and the A^q^-expressing strains whereas the response to the non-modified CII peptide was dramatically enhanced in the DR4 mice compared with the B10.Q. In addition, we show that the difference in the transgenic DR4-restricted T cell specificity to CII259-273 is not dependent on the degree of glycosylation of the collagen used for immunization.

**Conclusions:**

The present study provides important evaluation of CII-specific immune responses at different phases during CIA development as well as a comparative analysis between two CIA mouse models. We indicate significant differences in CII T cell and antibody specificities between the two strains and highlight a need for improved humanized B10.DR4 mouse model for rheumatoid arthritis.

## Introduction

Rheumatoid arthritis (RA) is one of the most common autoimmune diseases that generally affects peripheral joints and causes significant physical disability in around 1% of the human population worldwide. In spite of intensive investigation during the last decades RA etiology and pathogenesis are still unclear. Research with RA patients often faces impediments due to ethical reasons, complexity of affecting environmental factors and applied therapeutics. Thus, a standard approach to study disease mechanisms and causative agents is the utilization of animal models. A widely accepted model for RA is collagen type II-induced arthritis (CIA) in mice. The disease can be triggered in genetically susceptible strains bearing certain major histocompatibility complex class II (MHC II) haplotypes (H-2^q ^or H-2^r^) as well as in transgenic mice, expressing HLA-DR4 or DR1 RA-associated alleles [1-4]. CIA development depends on both T and B cell immune responses to collagen type II (CII) - the major constituent of joint cartilage, which is the main site of inflammation in CIA and also RA. Moreover, CII-specific T cell [5-7] and humoral [8,9] immunity has been detected in RA patients and hence CII is considered as a candidate autoantigen in RA pathogenesis.

Our group and others have extensively studied CII-directed immunity using the CIA model. We have identified a T cell immunodominant epitope from collagen type II (CII259-273) that plays a major role in CIA development in mice with H-2^q ^haplotype [10]. Subsequently, it was shown that this epitope also binds to human DR4 and DR1 molecules, associated with RA [11,12] and immunity to CII259-273 was detected also in RA patients [5,6,13]. Interestingly, the CII259-273 epitope could be posttranslationally modified (at position 264 and 270) by lysine hydroxylation and glycosylation. T cell responses to CII259-273 and its posttranslationally modified forms have been intensively studied in H-2^q^, as well as in DR-transgenic mice. So far, however, little is known about the magnitude and specificity of CII-directed T cell immunity over arthritis time course. It is unclear whether T cells display constant specificity to a certain form (native or posttranslationally modified) of the CII259-273 epitope or if T cell specificity shifts at different time points during disease development. Therefore, one of the main aims of the present study was to characterize the CII-specific T cell response in CIA over time analyzing four general stages of disease course: 1) the initial phase of CIA with no visible signs of disease, 2) the stage when joint inflammation become apparent, that is, disease onset, 3) the stage of established disease, and 4) the chronic phase of CIA. Two different mouse strains were used for this evaluation - B10.Q strain, expressing H-2^q ^MHC II haplotype on B10 genetic background, and a humanized transgenic strain, denoted as B10.DR4.Ncf1^*/*^, that expresses HLA-DR4 instead of mouse MHC II molecules on the same genetic background. The study of CII immunity in these two strains enables comparison between a classical mouse strain used in arthritis research and the recently developed transgenic strain that resembles the human situation more closely by expressing one of the most common HLA alleles in RA patients (HLA-DRB1*0401). In addition, a second aim of our study was to investigate the anti-CII antibody levels over the disease course and compare the response between the B10.Q and B10.DR4.Ncf1^*/* ^strain. Regarding the humoral response with significance for development of CIA and RA, several conserved epitopes from CII have been defined [14-16]. Research in mouse models indicated that antibodies to U1, C1 and J1 CII epitopes are arthritogenic and their levels correlate with development of chronic arthritis [17,18]. Thus, in order to evaluate anti-CII antibody fine specificity in B10.Q and DR4-transgenic mice we investigated the levels of C1, U1 and J1 epitope-specific antibodies. We found notable differences between B10.Q and DR4-transgenic mice regarding both humoral and T cell CII fine specificity. The majority of anti-CII antibodies in the two strains recognized the C1 epitope but in DR4-transgenic mice there was also sustained response to the U1 epitope. Interestingly, in general, anti-C1 antibody levels showed tendency for correlation with disease development while anti-U1 antibodies kept constant levels during CIA. T cell specificity also differed - in B10.Q mice there was predominant response to the glycosylated form of the CII259-273 epitope while in B10.DR4.Ncf1^*/* ^mice immunodominant was the native and the hydroxylated form of the epitope. We did not observe a shift in dominant epitope specificity at different stages of disease development in both mouse strains but the magnitude of T cell and antibody response showed dramatic increase at the acute stage of CIA.

## Materials and methods

### Antigens

Rat CII (rCII) was extracted from Swarm chondrosarcoma by limited pepsin digestion [19]. Human CII (huCII) was derived from hip joints and purified as previously described [19]. Both rCII and huCII proteins were dissolved in 0.1 M acetic acid and stored at 4°C. Three synthetic CII259-273 peptides were used: K264, representing the native non-modified rat CII259-273 (rCII259-273) T cell epitope (GIAGFKGEQGPKGEP), which have the same sequence in human, bovine and chick type II collagen; HOK264 - rCII259-273, containing (5R)-5-hydroxy-L-lysine at position 264; GalOK264 - rCII259-273, modified by linkage of β-D-galactopyranosyl residue to 5-hydroxy-L-lysine at position 264. The K264 and HOK264 peptides were produced by Schafer-N (Copenhagen, Denmark). The GalOK264 peptide was produced by Syngene (Bangalore, India). Three other synthetic triple helical CII peptides were used for evaluation of anti-CII antibody fine specificity: C1, corresponding to CII amino acid sequence from position 358-369 (CII358-369; GARGLTGRPGDA), U1, corresponding to CII494-504 (GLVGPRGERGF), and J1 that represents CII551-564 (GERGAAGIAGPK). The synthesis of these CII peptides has been previously described [14]. Concanavalin A (ConA) was purchased from Sigma-Aldrich (Munich, Germany), dissolved in PBS, sterile filtered and stored at -20°C until use.

### Animals

Two mouse strains were used in the present study both on B10 genetic background - B10.Q and B10.DR4.Ncf1^*/*^. The line B10.Q was initially provided by J. Klein (Tübingen, Germany) and maintained for more than two decades at the animal facility at the section for Medical Inflammation Research (Lund University, Sweden). The strain B10.DR4.Ncf1^*/* ^was generated by a cross between the B10.DR4 strain (transgenic mice that co-express HLA-DR4 and human CD4 receptor [20]) and the B10.Q Ncf1^*/* ^(a B10.Q strain with a mutation in the Ncf1 gene, leading to reduced reactive oxygen species production by the NOX2 phagocyte complex [21]). The resulting generation was intercrossed in order to yield homozygous DR4/humanCD4/H-2^-/- ^individuals with a mutation in the Ncf1 gene - the strain denoted as B10.DR4.Ncf1^*/*^. The animals were kept under standard conditions - controlled temperature, light and food regimen. All mice used for experiments were eight-to-twelve-weeks old, age and sex matched. Lund-Malmö laboratory animal ethics committee approved the conducted animal research.

### CIA induction and assessment

CIA was induced by immunization with 100 μ g of rat CII or human CII emulsified with 100 μ l complete Freund's adjuvant (CFA; Difco, Detroit, MI, USA). Three weeks postimmunization the animals were boosted with 50 μ g rCII or huCII emulsified with 50 μ l incomplete Freund's adjuvant (IFA; Difco). Evaluation of CIA was initiated two weeks after the primary immunization and the animals were examined two to three times per week. Disease manifestations were assessed using 60-point system with a maximal score of 15 points per paw [22].

### T cell *in vitro *assay

At day 15, 35, 50 and 65 after primary immunization five to ten mice were sacrificed; inguinal lymph nodes were isolated and passed through a 25-μ m nylon cell strainer (BD Discovery Labware, Franklin Lakes, NJ, USA). Single cell suspensions were washed twice with DPBS (Gibco, Life Technologies, Grand Island, NY, USA) and restimulated with CII259-273 peptides, conA and rCII or huCII. The cells were cultured in 96-well plates (Nalge Nunc International; Thermo Fisher Scientific, Rochester, NY, USA) for 96 h in 200 μ l Dulbecco's modified Eagle medium (DMEM; Gibco) supplemented with 10% heat-inactivated fetal calf serum (FCS; Gibco) and antibiotics (100 IU/ml penicillin and 100 μ g/ml streptomycin). At the end of the incubation period 150 μ l of the culture medium from each sample well were collected and frozen at -20°C until use. All samples were plated in triplicates.

### Cytokine ELISA

The collected culture medium samples were used to determine interferon-γ (IFN-γ) levels by sandwich ELISA. Ninety-six-well ELISA plates (Costar #3590; Corning Life Sciences, MA, USA) were coated with 50 μ l anti-mouse IFN-γ antibody (5 μ g/ml, clone R46-A2; Mabtech AB, Stockholm, Sweden) for 2 h at room temperature (RT) or overnight at 4°C. The plates were then washed with buffer and unspecific binding was blocked by 30 min incubation with 100 μ l/well 2% solution of non-fat dry milk. After that, the plates were washed again and incubated for 2 h at RT with 50 μ l culture medium from *in vitro *restimulated T cells. At the end of the incubation period washing was performed, 50 μ l biotinylated anti-mouse IFN-γ antibody (1 μ g/ml, clone AN18; Mabtech AB) was added to each sample well and incubated for 1 h at RT. After extensive washing Eu^3+^-labeled streptavidin was added to the plates and IFN-γ level in the samples was detected on a Wallac Victor 1420 multilabel counter (PerkinElmer, Waltham MA, USA) using the dissociation-enhanced lanthanide fluoroimmunoassay (DELFIA™) system (PerkinElmer). Recombinant mouse IFN-γ or standardized supernatant from conA-stimulated splenocytes were added to each ELISA plate as a positive control and standard.

### Anti-CII antibody measurement

Blood samples were taken from all experimental animals at day 15, 35, 50 and 65 after the primary immunization. Following centrifugation (6500 rpm for 20 min), serum was isolated from all samples, diluted in PBS (serum/PBS - 1/10 ratio) and stored at -20°C until use. The level of anti-CII antibodies in the samples was evaluated by ELISA. In brief, 96-well Costar plates (Corning) were coated overnight at 4°C with 10 μ g/ml phosphate solution of rCII or huCII. The plates were then washed and blocked for 30 min at RT using 2% non-fat milk solution. Following extensive washing, serum samples in serial dilutions were added to the plates and incubated for 2 h at RT. The plates were then washed and incubated with 1 μ g/ml solution of horseradish peroxidase (HRP)-conjugated anti-mouse immunoglobulin G (IgG) (heavy + light chain) antibody (Jackson ImmunoResearch Laboratories, West Grove, PA, USA) for 1 h at RT. After extensive washing, binding of anti-CII antibodies was revealed by the 2,2'-azino-bis(3-ethylbenzothiazoline-6-sulphonic acid (ABTS) system (Roche Diagnostics, Basel, Switzerland) and 405 nm-wavelength absorbance was detected using the Wallac Victor 1420 counter (PerkinElmer). Pooled polyclonal mouse serum with known anti-CII antibody concentration was included to each test plate and served as a positive control and standard.

For detection of epitope-specific anti-CII antibodies, Costar plates #3590 (Corning Life Sciences) were coated for 2 h at RT with 5 μ g/ml solution of synthetic triple-helical peptides (C1, U1 and J1). The subsequent steps of the assay are equivalent to the total anti-CII IgG ELISA, described in the previous paragraph. The levels of epitope-specific antibodies were determined using biotinylated anti-mouse IgG κ chain antibody (clone 187.1, incubated on the plates for 1 h at RT in 1 μ g/ml concentration). Detection of specific binding was carried out using Eu^3+^-labeled streptavidin and the DELFIA™ system (PerkinElmer) according to manufacturer's recommendations. Purified monoclonal antibodies to the three CII epitopes were used as a positive control and standard on all plates.

## Results

### CIA development in B10.Q and B10 DR4-transgenic mice

CIA was induced in order to compare arthritis development between the B10.Q strain and the DR4-transgenic mice and relate it to CII-specific immune responses at certain stages of disease. The strain B10.DR4 that co-expressed human CD4 and HLA-DRB*0401 and lacked endogenous expression of mouse MHC II molecules was resistant to CIA [23]. However, introducing a point mutation in the Ncf1 gene (yielding the strain B10.DR4.Ncf1^*/*^) led to susceptibility to arthritis [23]. The Ncf1 mutation results in expression of truncated Ncf1/p47phox protein that leads to reduced capacity for production of reactive oxygen species by the phagocytic NADPH complex [21]. Therefore, the B10.DR4.Ncf1^*/* ^is a more arthritis-susceptible DR4-expressing CIA model and individuals from this strain were used for the experiments presented in the current study.

**Figure 1:**
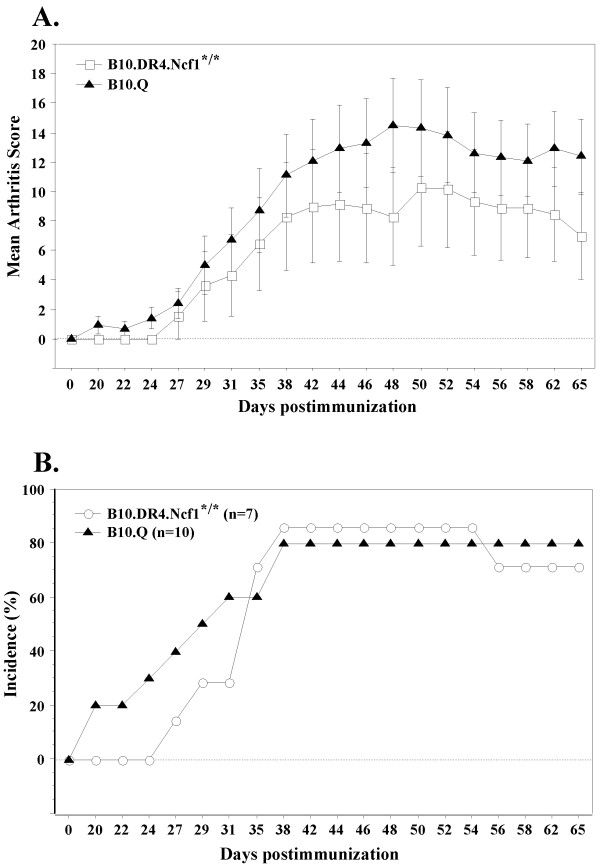
CIA disease course in B10.Q and B10.DR4.Ncf1^*/* ^mice*. Experimental animals were immunized with 100 μ g rCII in CFA (day 0) and three weeks later received a booster injection of 50 μ g rCII in IFA (day 21 postimmunization). CIA severity and incidence were evaluated two to three times weekly until the end of experiment (day 65 postimmunization). **(A) **Mean arthritis score in B10.Q (*n *= 10) and B10.DR4.Ncf1^*/* ^mice (*n *= 7). Data are presented as mean ± SE and include both arthritic and healthy animals. **(B) **CIA incidence in B10.Q and B10.DR4.Ncf1^*/* ^mice. * Data represent CIA assessment only in mice examined until the end of experiment. CIA data from mice killed for *in vitro *T cell assays are included in Table 1. CIA, collagen-induced arthritis; IFA, incomplete Freund's adjuvant; rCII, rat collagen type II.

B10.Q and B10.DR4.Ncf1^*/* ^mice were immunized with rCII and visual score of CIA development was conducted two to three times weekly until day 65 postimmunization. Compared to B10.Q mice, the disease onset in DR4-transgenics was observed at a later time point (Table 1, Figure 1). Arthritic mice from both strains demonstrated relatively similar mean disease severity at the four time points chosen for evaluation of CII-specific immune responses. B10.DR4.Ncf1^*/* ^mice displayed slightly lower disease incidence compared to B10.Q mice.

**Table 1:** Disease severity and incidence at specific time points during collagen-induced arthritis in B10

Strain	Mean day of onset †	Mean severity day 35 †	Mean severity day 50 †	Mean severity day 65 †	% Incidence (day 35 arthritic vs. total numberof mice)	% Incidence (day 50 arthritic vs. total number of mice)	% Incidence (day 65 arthritic vs. total numberof mice)
B10.Q	28 ± 3	13.4 ± 1.9	19.4 ± 2.2	14.1 ± 1.5	55 (11/20)	70 (14/20)	90 (18/20)
B10.DR4. Ncf1^*/*^	32 ± 1	16.4 ± 6	21 ± 5.6	9.8 ± 3.3	44.4 (8/18)	69.2 (9/13)	71.4 (5/7)

†Data represent mean values ± SE and include only arthritic animals.

### Longitudinal analysis of CII-specific T cell response in B10.Q and B10.DR4.Ncf1^*/* ^mice

To determine the CII-specific T cell response single cell suspensions from inguinal lymph nodes were restimulated *in vitro *with non-modified CII259-273 peptide (denoted as K264), hydroxylated CII259-273 (HOK264) and glycosylated CII259-273 peptide (GalOK264). Only peptides with modified lysine at position 264 were used because it was previously shown that this is the key amino acid residue for CII-specific T cell recognition in H-2^q ^and HLA-DR4 mouse models [23,24]. Samples from B10.Q and B10.DR4.Ncf1^*/* ^mice were collected at four time points after CIA induction: day 15, day 35, day 50 and day 65, all of them corresponding to different stage of disease development. T cell activation in response to the CII peptides was ascertained by detection of Th1-specific IFN-γ levels in the culture medium following restimulation. It is known that other T cell subpopulations, that is, IL-17-secreting T cells [25], are also present during CIA. We recently detected Th17 cells in B10.DR4.Ncf1^*/* ^mice [23], and showed that their specificity to CII259-273 is mainly to the unmodified epitope. However, our present study was concentrated on the Th1 cell subtype, measuring IFN-γ levels, because CIA is generally considered as a Th1-mediated disease [26] and susceptibility to CIA is associated with certain MHC II allotype.

**Figure 2:**
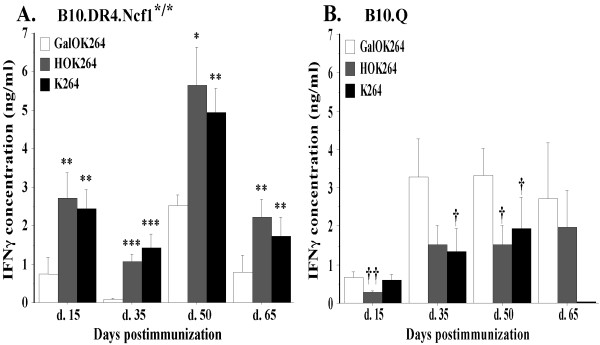
**T cell specificity to the CII259-273 epitope and its posttranslationally modified forms**. B10.Q and B10.DR4.Ncf1^*/* ^mice were immunized with 100 μ g CII in CFA (day 0) and boosted 21 days later with 50 μ g CII in IFA. Five to ten mice per group were sacrificed at day 15, 35, 50 and 65 postimmunization. Single cell suspensions from collected inguinal lymph nodes were incubated *in vitro *with collagen peptides or (conA) for 96 h. Culture supernatant was then used for measurement of INF-γ content. **(A) **T cell recall response in B10.DR4.Ncf1^*/* ^strain. The symbol '*' marks responses that are significantly higher than the GalOK264 response. *, *P *<.05; **, *P *<.01; ***, *P *<.001. Mean IFN-γ values for the conA stimulated positive control: d. 15 - 10.276 ± 0.899 ng/ml; d. 35 - 4.386 ± 0.269 ng/ml; d. 50 - 15.915 ± 1.349 ng/ml; d. 65 - 7.996 ± 1 ng/ml. **(B) **CII-specific T cell response in B10.Q mouse strain. The symbol '†' marks significantly lower responses compared to GalOK264 response. †, *P *<.05; ††, *P *<.01. Mean IFN-γ values of the positive control: d. 15 - 1.927 ± 0.321 ng/ml; d. 35 - 7.004 ± 0.231 ng/ml; d. 50 - 4.569 ± 0.631 ng/ml; d. 65 - 2.4 ± 0.3 ng/ml. Background values (IFN-γ levels secreted by cells cultured in standard medium without CII peptides or conA) are subtracted from the presented data. All data represent mean ± SE of triplicates. CFA, complete Freund's adjuvant; con A, concanavalin A; CII, collagen type II; IFA, incomplete Freund's adjuvant; IFN, interferon.

Figure 2 illustrates the results from the measurement of CII-specific T cell responses. Confirming previous findings [23,27], the data clearly shows that in the B10.Q strain the glycosylated form of CII259-273 (GalOK264) is immunodominant while in B10.DR4.Ncf1^*/* ^mice immunodominant is the non-modified epitope (K264) and also the hydroxylated form of the epitope (HOK264). The main reason for this difference is that the response to the non-modified and the hydroxylated peptide is dramatically enhanced in the DR4 mice. The response to the GalOK264 peptide on the other hand is also increased at the established disease phase or in arthritic mice.

Nevertheless, the T cell response to the immunodominant epitope in both strains was maintained during all stages of CIA - at all studied CIA time points B10.Q mice responded strongest against GalOK264 while B10.DR4.Ncf1^*/* ^strain responded strongest against K264 and HOK264. We observed elevated level of recognition of GalOK264 in B10.DR4.Ncf1^*/* ^at day 50 postimmunization but still the magnitude of this response was significantly lower compared to K264 and HOK264. On the other hand, B10.Q mice displayed T cell response to the non-modified CII259-273 epitope during the early stage of CIA development (day 15 postimmunization) while the recognition of HOK264 was insignificant. However, at the later time points (day 35, day 50, day 65) of disease development we detected higher response to the hydroxylated form of CII259-273. This tendency is markedly pronounced on day 35 and day 65 postimmunization when we could not find significant difference between the response to HOK264 and the response to the immunodominant GalOK264 epitope. This result suggests that T cell recognition of HOK264 in B10.Q mice tends to correspond to manifestation of clinical signs of arthritis while K264-specific T cells could play a role in the initial phase of disease. In general, the level of CII-specific T cell activation in B10.Q mice showed tendency for correlation with disease clinical score - the lowest magnitude of response to CII259-273 was observed at day 15 when there were no visible signs of arthritis but the level of CII-specific T cell activation increased dramatically at the time points when arthritis symptoms are evident. Interestingly, this was not the case with B10.DR4.Ncf1^*/* ^strain (Figure 2, A). At the initial phase of disease (day 15 postimmunization) we detected a strong IFN-γ response against K264 and HOK264 while at day 35 postimmunization the level of IFN-γ secretion following restimulation with K264 and HOK264 peptides was lower. Fifty days after CII immunization when most experimental animals showed the highest CIA severity, we detected also the highest level of IFN-γ concentration in response to CII259-273. And expectedly, at day 65 postimmunization the magnitude of CII-specific T cell responses decreased in line with the slightly reduced CIA severity (Table 1, Figure 1, A).

### Longitudinal analysis of CII-directed humoral response in B10.Q and B10.DR4.Ncf1^*/* ^mice

**Figure 3:**
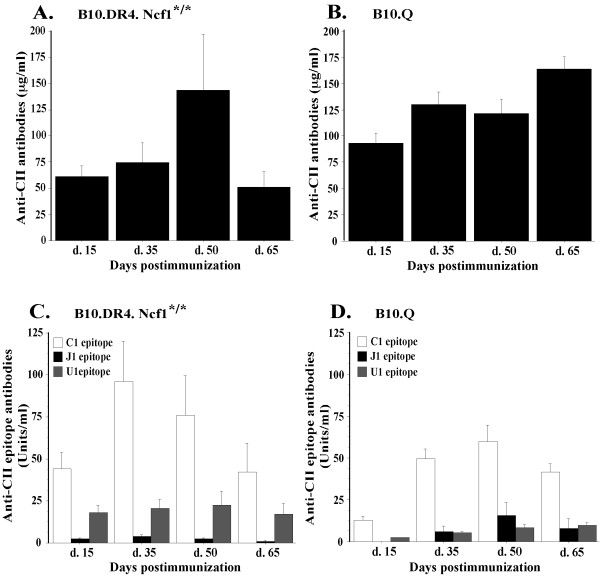
**CII-specific antibody response at different time points after CIA induction**. Blood samples were taken from B10.Q and B10.DR4.Ncf1^*/* ^mice at four time points after rCII immunization (day 15, 35, 50 and 65). Extracted sera were used to assess the levels of anti-CII antibodies, anti-C1, anti-U1 and anti-J1 epitope-specific antibodies. **(A) **Total anti-CII antibody concentration in sera of B10.DR4.Ncf1^*/* ^mice (number of animals per group: day 15 - *n *= 22, day 35 - *n *= 16, day 50 - *n *= 10, day 65 - *n *= 5). **(B) **Total anti-CII antibodies concentration in B10.Q mice (*n *= 20 per each time point). **(C) **Anti-C1, anti-U1 and anti-J1 antibody levels, measured in B10.DR4.Ncf1^*/* ^mouse strain (number of animals per group: day 15 - *n *= 22, day 35 - *n *= 16, day 50 - *n *= 10, day 65 - *n *= 5). **(D) **Levels of anti-CII epitopes (C1, U1, J1) antibodies in B10.Q mice (*n *= 20 per each time point). Results indicate mean ± SE. CIA, collagen-induced arthritis; CII, collagen type II.

CIA development depends on both T and B cell responses. Thus, together with CII-specific T cell recognition we analyzed also anti-CII antibody levels during different stages of CIA. Concentration of total anti-CII IgG antibodies in B10.Q and B10.DR4.Ncf1^*/* ^mice was measured at four time points during CIA (day 15, day 35, day 50, day 65 postimmunization). Overall, antibody titers in DR4-transgenic mice showed a tendency for correlation with CIA development (Figure 3, A). Significant level of anti-CII IgG was detected already at day 15 following immunization preceding manifestation of disease visual symptoms. At day 35, when around 40% of the experimental animals displayed clinical signs of CIA, anti-CII antibodies titer increased. The highest concentration of CII-specific antibodies was measured at day 50 (established CIA) - the stage when the majority of the mice were sick, many of them suffering severe arthritis. At the end of the experiment, day 65 postimmunization, antibody concentration was still sustained but decreased in line with reduced CIA severity in most of the experimental animals. Compared to B10.DR4.Ncf1^*/* ^mice, the B10.Q strain displayed higher serum titers of anti-CII antibodies (Figure 3, B). Again, elevated antibody concentration was detected before disease onset (day 15 postimmunization) and it increased further together with the manifestation of clinical signs of CIA (day 35 and day 50). Interestingly, B10.Q mice showed the highest anti-CII antibodies level at the end of experiment (day 65 postimmunization). Presumably, this result could be due to the high incidence of sick animals (90% in total, Table 1) and also more severe disease in comparison with B10.DR4.Ncf1^*/* ^(Table 1, Figure 1, A).

Together with total anti-CII antibody titers, epitope-specific antibody levels were evaluated. In CIA and also in RA CII-specific antibodies recognize and bind certain conserved epitopes [14-16]. To determine anti-CII antibodies fine specificity in a longitudinal manner and compare it between B10.Q and B10.DR4.Ncf1^*/* ^mice, we measured anti-U1, anti-C1 and anti-J1 antibody titers at the four chosen time points during CIA development. As shown on Figure 3 (C, D) the majority of epitope-specific anti-CII antibodies recognized the C1 epitope. This specificity dominated during all stages of CIA development in both experimental strains. The highest levels of anti-C1 antibodies were detected on day 35 and day 50 postimmunization in line with manifestation of clinical signs of CIA. In addition, B10.Q mice showed a low response to U1 and J1 epitopes that was most prominent at the time points when arthritis symptoms are present. The B10.DR4.Ncf1^*/* ^mice exhibited a prominent response also to U1 epitope, while recognition of J1 epitope was negligible in both strains.

### Arthritic B10.Q and B10.DR4.Ncf1^*/* ^mice display CII-specific immune responses with significantly higher magnitude

**Figure 4:**
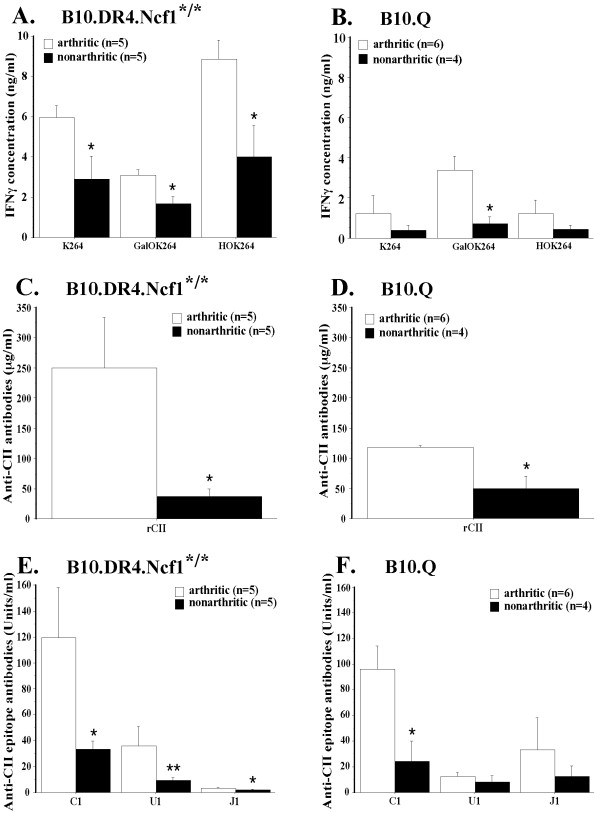
**Comparison of CII-specific T cell and antibody responses in arthritic vs. nonarthritic animals (day 50 postimmunization)**. **(A) **IFN-γ recall response to CII259-273 peptides, measured in sick and healthy B10.DR4.Ncf1^*/* ^mice. Mean positive control values: arthritic mice - 17.294 ± 1.274 ng/ml; nonarthritic animals - 14.696 ± 2.266 ng/ml. **(B) **CII-specific T cells response in arthritic vs. nonarthritic B10.Q mice. Mean IFN-γ values for the positive control: arthritic animals - 5.199 ± 0.819 ng/ml; nonarthritic mice - 3.624 ± 0.903 ng/ml. Background INF-γ levels were subtracted from all T cell responses data. **(C) **and **(D) **Anti-CII antibody concentrations in arthritic vs. nonarthritic B10.DR4.Ncf1^*/* ^and B10.Q mice, respectively. **(E), (F) **Levels of antibodies, specific to C1, U1 and J1 CII epitopes in B10.DR4.Ncf1^*/* ^and B10.Q sick and healthy mice, respectively. All data display mean ± SE. *, *P *<.05; **, *P *<.01; ***, *P *<.001. CII, collagen type II; IFN, interferon.

During a standard CIA experiment with B10.Q and B10.DR4.Ncf1^*/* ^mice disease symptoms are well pronounced around day 50 postimmunization. Usually, the animals that have not shown CIA clinical signs until this time point do not develop disease. Therefore, we chose this stage of the experiment for comparison of CII-specific immune responses between arthritic and healthy B10.Q and B10.DR4.Ncf1^*/* ^individuals. The results, illustrated in Figure 4, show a clear tendency for significantly stronger CII-directed reactivity in sick animals in both strains. We even found markedly increased T cell response to GalOK264 peptide in B10.DR4.Ncf1^*/* ^arthritic mice (Figure 4, A). This data suggest a certain role, although not a dominant one, for the glycosylated CII-259-273 epitope in CIA in the DR4-transgenic model. In the case of B10.Q mice, significant difference between arthritic and healthy animals was present only for the T cell reactivity to GalOK264 peptide (Figure 4, B). When looking at the humoral response, we could see a marked contrast between arthritic and non-arthritic animals in both strains on the level of total anti-CII antibodies (Figure 4, C and 4D), as well as anti-C1 antibodies (Figure 4, E and 4F). B10.DR4.Ncf1^*/* ^arthritic mice had also significantly higher levels of anti-U1 and anti-J1 antibodies.

### T cell response fine specificity in B10.DR4.Ncf1^*/* ^mice is not dependent on the glycosylation level of administrated CII

**Figure 5:**
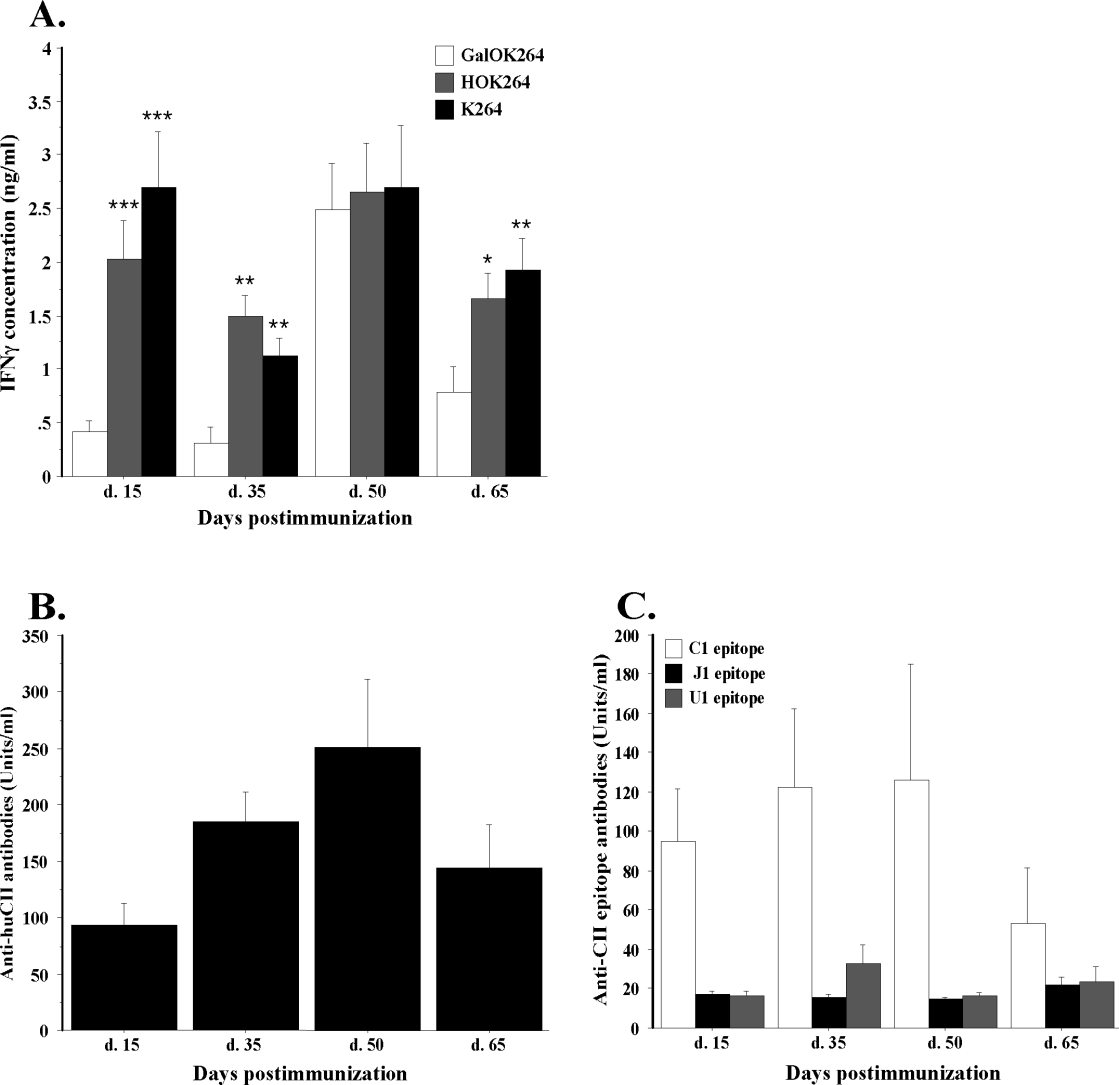
T cell specificity to the CII259-273 epitope and its posttransationally modified forms in B10.DR4.Ncf1^*/* ^mice following immunization with huCII. Experimental animals were immunized with 100 μ g huCII emulsified in CFA at day 0. Twenty-one days later the mice received a booster immunization of 50 μ g huCII in IFA. At four particular time points after immunization (day 15, 35, 50, 65) five mice per group were sacrificed and inguinal lymph nodes were harvested for further analysis. **(A) **T cell recall responses in huCII-immunized B10.DR4.Ncf1^*/* ^mice following *in vitro *restimulation with CII259-273 peptides. Mean IFN-γ values for the conA positive control at each time point: d. 15 - 2.884 ± 0.313 ng/ml; d. 35 - 2.137 ± 0.485 ng/ml; d. 50 - 4.732 ± 0.53 ng/ml; d. 65 - 4.632 ± 0.291 ng/ml. Responses that are significantly higher than the GalOK264 response were marked with '*'. Background IFN-γ values are subtracted from the presented data. **(B) **Total CII-specific antibody levels in B10.DR4.Ncf1^*/* ^mice immunized with huCII. **(C) **CII epitope-specific antibody levels B10.DR4.Ncf1^*/* ^mice immunized with huCII. Data designate mean ± SE. *, *P *<.05; **, *P *<.01; ***, *P *<.001. CFA, complete Freund's adjuvant; con A, concanavalin A; (hu)CII, (human) collagen type II; IFA, incomplete Freund's adjuvant; IFN, interferon.

The present study demonstrates a remarkable difference between the B10.Q and the B10.DR4.Ncf1^*/* ^model on the level of CII-directed T cell specificity. B10.Q T cells generally react against the glycosylated CII259-273 epitope, while T cells in DR4-transgenics predominantly recognize the native and hydroxylated CII259-273 epitope. One possible explanation for this contrast could be the presence of second lysine residue at position 270 in CII259-273 that could play a major role in T cell recognition in the context of DR4 restriction. However, our previous studies have shown that this is not the case and that K264 is the main T cell receptor interaction point [23]. Another reason could be the collagen type II, used for immunization of experimental animals and its level of glycosylation. If the immunizing CII displays higher extent of glycosylation then it could induce a stronger GalOK264 response. The rCII that is routinely used for immunization is derived from Swarm chondrosarcoma - a tumor cell line that produces collagen molecules with both hydroxylated and glycosylated residues [28]. In contrast, CII derived from normal cartilage is more uniformly glycosylated as determined in rats and humans [28]. Therefore, to test whether the glycosylation extent of CII could influence T cell reactivity, we immunized B10DR4.Ncf1^*/* ^mice with huCII and evaluated CII-specific immune responses during CIA. The results from this experiment, however, confirmed the immunodominant status of K264 and HOK264 for CII-specific T cell recognition in DR4-transgenic mice (Figure 5, A). The only stage of CIA development when we could see significant GalOK264 recognition was day 50 postimmunization, again, suggesting cartilage immune recognition leading to epitope spreading in line with the peak of disease severity. The magnitude of T cell response also followed the same trend as the B10.DR4.Ncf1^*/* ^mice immunized with rCII - strong T cell reaction at day 15 and 50 postimmunization, and lower responses at day 35 and 65. Expectedly, total anti-CII antibody responses were also similar and anti-C1 antibodies predominated the epitope-specific response (Figure 5, B and C).

## Discussion

In both RA and CIA joint cartilage is affected by massive inflammation that drives destruction of the tissue. Immune responses against CII, the main synovial component, take part in this process. During arthritis, CII is degraded to small peptides that are presented to T cells. Previously, we identified the immunodominant epitope CII259-273 [10] in which the K264 residue plays a key role for CII-specific T cell recognition in mouse models expressing either A^q ^or HLA-DR4 MHC II molecules [23,24]. Furthermore, we have demonstrated that K264 is glycosylated in normal human and rat joint cartilage while in arthritic cartilage the CII259-273 epitope is present in both non-modified and glycosylated form (K264 and GalOK264, respectively) [28]. This finding raised an important question for the role of K264 glycosylation/deglycosylation state in arthritis development. Therefore, the present study aimed to investigate CII-specific T cell responses at different time points of CIA in order to clarify whether there is a shift in T cell recognition of CII259-273 and its posttranslationally modified forms during disease development. We have chosen to focus on Th1 response in this study as CII-specific T cell response in CIA is dominated by Th1 cells [26], which produce IL-2 and IFN-γ and could thus drive the production of complement-fixing CII-specific IgG2a, an important component in the pathogenesis of CIA [29]. Interestingly, no shift in T cell recognition was observed during the phases investigated. In general, B10.DR4.Ncf1^*/* ^mice displayed dominant T cell reactivity to the non-modified CII259-273 and also to the hydroxylated form of the epitope. The dominant B10.Q T cell response was directed against the glycosylated CII259-273 epitope. An exception to this rule was seen only at the initial stage of CIA in B10.Q mice when a relatively equal T cell reactivity to non-modified CII259-273 and to the glycosylated peptide was detected. One possible reason for this discrepancy could be the CII used for immunization. This protein contains both non-modified and glycosylated CII259-273 epitopes since it is extracted from Swarm chondrosarcoma, which do not uniformly glycosylate the CII molecule [28]. Thus, it is to be expected that the mice would initially react against both glycosylated and non-modified epitope since following immunization both epitopes are picked up from the CII molecule and presented by T cells. Such tendency, although not that strong, is also evident in B10.DR4.Ncf1^*/* ^mice where, in addition to the strong K264 and HOK264 response, we detected certain level of response to the GalOK264 peptide. Moreover, previous studies on T cell responses at an earlier time point following immunization showed even more pronounced response against the glycosylated epitope in DR4 mice (Dzhambazov *et al*., unpublished data). Therefore, we assume that presentation and recognition of both non-modified and glycosylated CII259-273 following immunization occurs earlier in DR4-transgenic mice compared to B10.Q mice. That would explain also the very high magnitude of response to K264 and HOK264 epitopes detected in B10.DR4.Ncf1^*/* ^mice already at day 15 postimmunization.

The lack of uniform glycosylation level of Swarm-derived rCII could also be the reason for stronger T cell response to non-modified CII259-273 epitope in B10.DR4.Ncf1^*/* ^mice. To test this hypothesis, a group of DR4 mice with human CII was immunized with a protein extracted from normal human cartilage that is known to contain exclusively glycosylated CII259-273 [28]. The results, however, could not clearly confirm our hypothesis. Again, we detected a dominant T cell response to K264 and HOK264 epitopes. The only time point when we identified a significant GalOK264 response was day 50 postimmunization. At this time point, we detected a higher reactivity to the glycosylated epitope also in mice immunized with Swarm chondrosarcoma-derived rCII. During this stage of disease, the stronger response to the GalOK264 peptide could be attributed to epitope spreading in T cell specificity due to the acute inflammation process and developed immunity to the endogenous CII. Similarly, certain response to the other forms of CII259-273 epitope, in addition to the immunodominant one, was detected also in B10.Q mice at later time points of CIA (day 35, day 50, day 65).

The T cell responses in RA patients carrying HLA-DRB*04 alleles show a diverse pattern of CII259-273 epitope recognition, that is, toward both non-modified and glycosylated CII259-273; only few patients exclusively responded to K264 but most of them to GalOK264 [5,6]. A longitudinal analysis of CII-specific T cell responses showed a consistent reactivity but variable magnitude and specificity of the response over time [6]. Concerning the magnitude of T cell reactivity our results are in line with the RA patients' data since we could not see a correlation between disease visible signs (disease severity) and the level of IFN-γ secreted in response to CII259-273 stimulation in DR4-expressing mice. Also, in concordance with the human patients' data our results demonstrate significantly higher CII responses in arthritic compared to healthy individuals. However, when looking at T cell response specificity the results from RA patients are in contrast with our findings in DR4-transgenic mice where we could demonstrate a very strong response to the K264 epitope. This discrepancy raises an important question - whether the DR4-transgenic mouse model could accurately resemble the human disease setting and whether there is a need for improving the humanized model. Indeed, the standard transgenic technology implies insertion of an unknown number of transgene copies into the mouse genome and also the site/sites of transgene insertion is/are uncertain, which in turn could affect the physiological expression of the transgene itself. Interestingly, the more physiologic A^q ^mouse model resembles the CII response in RA better than the more artificial but humanized DR4 mouse.

CII-specific immunity in CIA and RA include both T and B cell responses. Thus, we included to our studies evaluation of anti-CII antibody levels during CIA. The longitudinal analysis of total CII-specific titers in B10.DR4.Ncf1^*/* ^showed a trend for correlation with disease severity but in B10.Q mice we detected a different pattern of antibody levels during CIA with day 65 (about the end of the CIA acute phase characterized by slightly decreased disease severity) showing the highest anti-CII IgG concentration. It is likely that the high antibody levels at day 65 is triggered by an endogenous response to cartilage CII and could precede and contribute to another peak of disease severity at a later time point. On the other hand, it has been shown that high anti-CII antibody titers do not exclusively lead to more severe disease [30]. The total CII-specific levels are polyclonal and include clones that do not contribute to arthritis development [14]. Thereby, an epitope-specific antibody response will be expected to show a better tendency for correlation with disease. Three dominant conserved CII epitopes - C1, U1, and the J1 epitope, can be recognized both in CIA and RA [14,15]. Our experiments included evaluation of epitope-specific antibody responses in B10.Q and B10.DR4.Ncf1^*/* ^mice and indicated a dominant response to the C1 epitope during the different phases of CIA. Anti-C1 antibody levels showed a positive relation to disease clinical score - the highest titers were detected during acute stages of CIA while at day 65 postimmunization C1-specific antibodies decreased in both mouse models. B10.DR4.Ncf1^*/* ^mice showed very low level of recognition of J1 epitope but a sustained response to U1 epitope. Interestingly, anti-U1 antibody levels were relatively constant at all studied CIA time points. In fact, previous studies on U1-specific monoclonal antibody have shown that it binds and destabilizes cartilage independent of inflammation [31]. It is likely that this mechanism of action is the reason for a lack of increase of U1-specific antibody titers at the acute stage of disease. However, additional experiments are needed in order to clarify whether this result could be due to non-optimal physiological setting in the transgenic model. This study was limited to three immunodominant CII epitopes. Despite the lack of epitope shift during the various disease phases investigated one cannot exclude involvement of additional CII epitopes during different stages of CIA, an ongoing study (Lindh *et al*., unpublished data).

## Conclusions

The present study provides important evaluation of CII-specific immune responses at different phases during CIA development as well as a comparative analysis between two CIA mouse models. We show evidence for dominant and specific recognition of CII T and B cell epitopes during CIA in both studied mouse strains. We did not observe a shift in CII epitope specificity at different stages of disease development. A trend for epitope spreading during acute stage of CIA was evident in both B10.Q and B10.DR4.Ncf1^*/* ^mice. Significant differences in CII T cell and antibody specificities between the two mouse models were indicated. Importantly, we also highlight a need for improved humanized B10.DR4 mouse model as the transgenic expression of human MHC II leads to a skewing of the T cell response towards the non-glycosylated form of the CII259-273 epitope.

## Abbreviations

ABTS: 2,2'-azino-bis(3-ethylbenzothiazoline-6-sulphonic acid); CIA: collagen-induced arthritis; CII: collagen type II; conA: concanavalin A; DELFIA: dissociation-enhanced lanthanide fluoroimmunoassay; (D)PBS: (Dulbecco's) phosphate-buffered saline; ELISA: enzyme-linked immunosorbent assay; huCII: human collagen type II; IFN: interferon; IgG: immunoglobulin G; IL: interleukin; MHC II: major histocompatibility complex class II; RA: rheumatoid arthritis; rCII: rat collagen type II; RT: room temperature.

## Competing interests

The authors declare that they have no competing interests.

## Authors' contributions

All authors participated in drafting the article or critically revising it for important intellectual content. TB, IL and BD were involved in the acquisition of data. BD and RH conceived and designed the study. TB, IL, BD, JB and RH analyzed and interpreted the data. RH supervised the project. All authors approved the final version of the manuscript.
